# Glymphatic function from diffusion-tensor MRI to predict conversion from mild cognitive impairment to dementia in Parkinson’s disease

**DOI:** 10.1007/s00415-024-12525-8

**Published:** 2024-06-24

**Authors:** Huize Pang, Juzhou Wang, Ziyang Yu, Hongmei Yu, Xiaolu Li, Shuting Bu, Mengwan Zhao, Yueluan Jiang, Yu Liu, Guoguang Fan

**Affiliations:** 1 Department of Radiology, First Affiliated Hospital of China Medical University, Shenyang, Liaoning China; 2 School of Medicine, Xiamen University, Xiamen, Fujian China; 3 Department of Neurology, First Affiliated Hospital of China Medical University, Shenyang, Liaoning China; 4 MR Research Collaboration, Siemens Healthineers, Beijing, China

**Keywords:** Parkinson’s disease, Glymphatic function, Perivascular space, Cognitive impairment, Dementia conversion

## Abstract

**Background:**

Although brain glymphatic dysfunction is a contributing factor to the cognitive deficits in Parkinson’s disease (PD), its role in the longitudinal progression of cognitive dysfunction remains unknown.

**Objective:**

To investigate the glymphatic function in PD with mild cognitive impairment (MCI) that progresses to dementia (PDD) and to determine its predictive value in identifying individuals at high risk for developing dementia.

**Methods:**

We included 64 patients with PD meeting criteria for MCI and categorized them as either progressed to PDD (converters) (*n* = 29) or did not progress to PDD (nonconverters) (*n* = 35), depending on whether they developed dementia during follow-up. Meanwhile, 35 age- and gender-matched healthy controls (HC) were included. Bilateral diffusion-tensor imaging analysis along the perivascular space (DTI-ALPS) indices and enlarged perivascular spaces (EPVS) volume fraction in bilateral centrum semiovale, basal ganglia (BG), and midbrain were compared among the three groups. Correlations among the DTI-ALPS index and EPVS, as well as cognitive performance were analyzed. Additionally, we investigated the mediation effect of EPVS on DTI-ALPS and cognitive function.

**Results:**

PDD converters had lower cognitive composites scores in the executive domains than did nonconverters (*P* < 0.001). Besides, PDD converters had a significantly lower DTI-ALPS index in the left hemisphere (*P* < 0.001) and a larger volume fraction of BG-PVS (*P* = 0.03) compared to HC and PDD nonconverters. Lower DTI-ALPS index and increased BG-PVS volume fraction were associated with worse performance in the global cognitive performance and executive function. However, there was no significant mediating effect. Receiver operating characteristic analysis revealed that the DTI-ALPS could effectively identify PDD converters with an area under the curve (AUC) of 0.850.

**Conclusion:**

The reduction of glymphatic activity, measured by the DTI-ALPS, could potentially be used as a non-invasive indicator in forecasting high risk of dementia conversion before the onset of dementia in PD patients.

## Introduction

Parkinson’s disease (PD) is a chronic neurodegenerative disorder characterized by motor dysfunction, with several nonmotor symptoms also being recognized as being integral to PD [1]. Cognitive impairment is a significant nonmotor symptom, with PD dementia being a late complication that affects 75–90% of patients with a disease duration of 10 years or more [2]. Mild cognitive impairment (MCI) is considered an intermediate stage on the continuum between normal cognitive function and dementia, and is regarded as a risk factor for dementia [3]. However, there is a substantial variability in cognitive profile and decline among PD patients, with some progressing to dementia, while others remaining stable or reverting to normal cognition [3]. Hence, stratifying PD patients with MCI based on their risk for dementia development is essential to tailor therapeutic strategies according to their predicted risk. Understanding the predicted risk can also provide valuable information on the underlying pathophysiology of cognitive decline in PD patients.

The maintenance of brain homeostasis relies on the removal of waste from the central nervous system. Studies by Iliff et al. first highlighted the role of the glymphatic system in neurodegenerative diseases, showing that waste products associated with Alzheimer’s disease (AD), such as amyloid beta protein and tau oligomers, are transported and cleared from the brain through the glymphatic system [4]. Previous animal studies have verified that suppressing glymphatic fluid transport could result in pathologic amyloid-β and α-synuclein deposits, which are associated with AD and PD pathology. In turn, restoring the glymphatic activity could be a promising therapeutic target for addressing cognitive decline in AD and PD patients [5–7]. Nevertheless, investigating glymphatic function in humans with neurodegenerative disease is challenging, given the difficulty of accurately measuring glymphatic function in vivo.

Gadolinium-based contrast agent (GBCA) enhanced magnetic resonance imaging is the most commonly applied method for assessing glymphatic function in vivo as it traces the delayed clearance of the agent as evidence of glymphatic dysfunction [8]. However, its clinical application is limited due to the need for contrast agent injection. Noninvasive MRI-based methods, namely diffusion-tensor imaging analysis along the PVS (DTI-ALPS) index, have been introduced as promising methods that do not require contrast injection and allow for the indirect evaluation of perivascular structure activity. Prior research has shown that the DTI-ALPS index, serving as an indirect indicator for evaluating glymphatic function, exhibits excellent reliability and reproducibility [9]. Besides, enlarged perivascular space (EPVS) is reported to be caused by metabolic waste obstruction, serving as an additional index for assessing glymphatic function [10]. Using the DTI-ALPS method, previous researchers have identified the role of glymphatic dysfunction in PD at different stages [11–19], as well as patients with isolated eye movement behavior disorder (RBD) [18, 20]. They have also reported correlations between DTI-ALPS and cognitive function [11], as well as motor performance [11, 21, 22]. Among the aforementioned studies, only one longitudinal research has explored the glymphatic activity in the phenoconversion risk of patients with RBD convert to α-synucleinopathies [20]. To the best of our knowledge, there is currently no study that has evaluated the role of glymphatic function in vivo in the risk of conversion to dementia in patients with PD and MCI.

Herein, in this longitudinal study, in the cohort of PD with MCI patients, using the DTI-ALPS index, we aimed to explore: (1) whether glymphatic function played a role in the risk of conversion to dementia in patients with PD and MCI. (2) the correlation between the DTI-ALPS index and EPVS with cognitive performance. (3) the potential of glymphatic function as an indicator to predict the dementia conversion in MCI stage. We hypothesized that a disrupted glymphatic system might be correlated with a high risk of developing dementia, and that glymphatic function could serve as a predictive indicator for predicting dementia conversion in PD patients with MCI.

## Materials and methods

### Participants

**Fig. 1:**
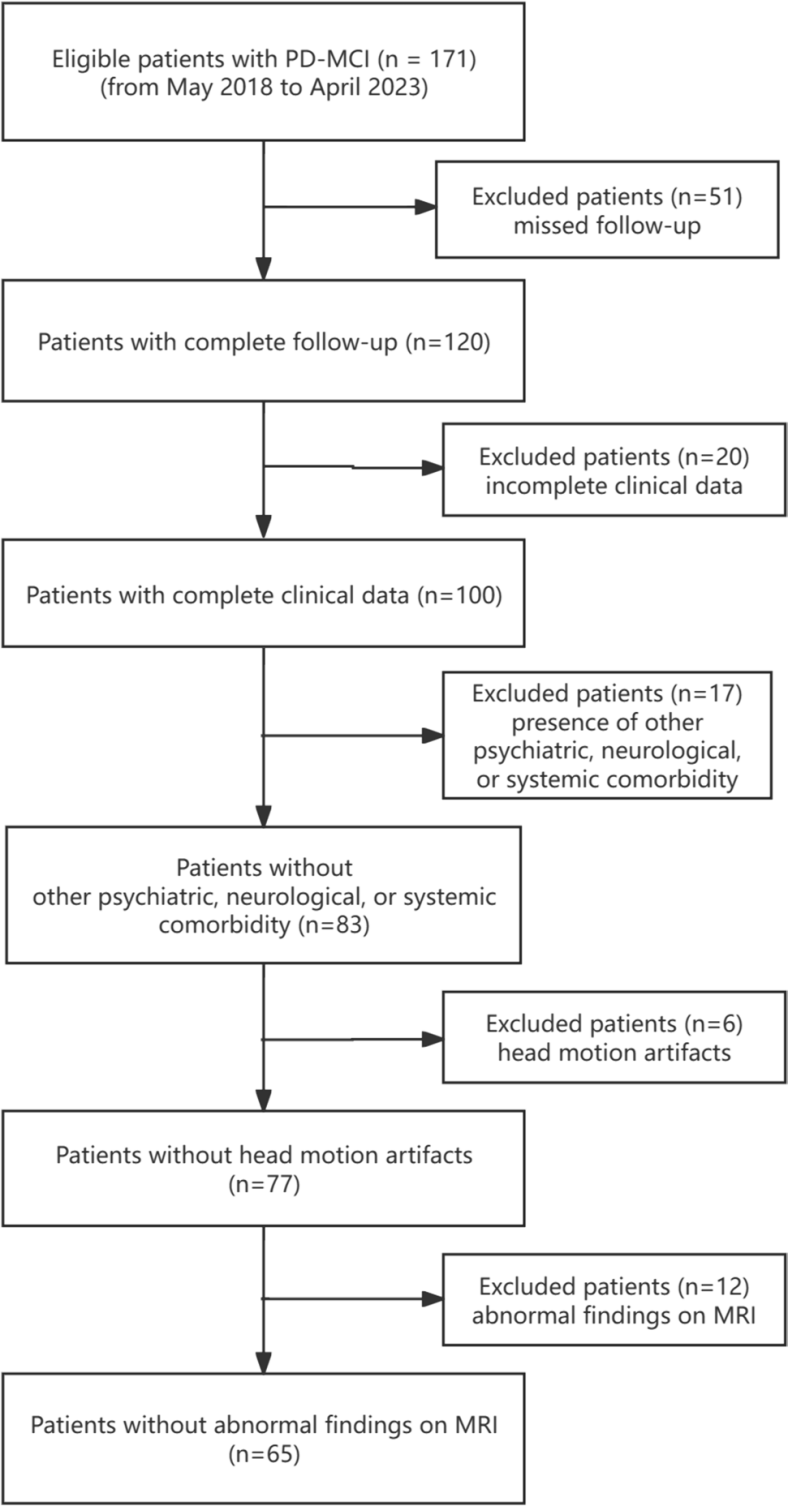
Flowchart of patient inclusion and exclusion criteria. *PD-MCI* Parkinson’s disease with mild cognitive impairment

From May 2018 to April 2023, a total of 171 PD patients with MCI were initially enrolled from the First Affiliated Hospital of China Medical University. All PD patients were diagnosed in accordance with the international Parkinson and Movement Disorder Society (MDS) Clinical Diagnostic Criteria for PD. PD patients with MCI were diagnosed using the MDS diagnostic criteria (level I), which includes a MoCA score < 26 or 2 or more cognitive tests with scores > 1.5 standard deviation below the standardized mean. All patients with PD and MCI were followed up for at least 1 year, with neuropsychologic tests. Exclusion criteria included missed follow-up (*n* = 51), incomplete clinical data (*n* = 20), presence of other significant psychiatric, neurological, or systemic comorbidity (*n* = 17), head motion artifacts (*n* = 6), and abnormal findings on conventional MRI (*n* = 12). A total of 64 PD patients with MCI were ultimately included (Fig. 1). Meantime, 35 age- and sex-matched healthy controls were included in this study. PD patients with MCI were divided into PDD converters and nonconverters according to the subsequent development of dementia during follow-up. The diagnosis of PDD was established based on the MDS criteria for probable PDD [23]. This study received approval from the Ethics Committee of China Medical University, and all participants signed written informed consent form.

Global cognitive function was measured using the Mini-Mental State Examination (MMSE) and Montreal Cognitive Assessment (MoCA) at the baseline and the follow-up timeline. Cognitive function was further examined in five subdomains: executive, attention/working memory, memory, visuospatial, and language subdomains. The cognitive tests were performed in a calm, light, and quiet environment under the guidance of experienced practitioners, without any interference. In addition, the Unified Parkinson’s Disease Rating Scale (UPDRS) and the Hoehn and Yahr (H–Y) stage were measured to evaluate the severity of movement impairment.

### Imaging techniques

#### MRI scanner settings

Imaging data were acquired using a 3.0 T MRI scanner (Magnetom Verio, Siemens Healthineers) with a 32-channel head coil in the Department of Radiology. The MRI scans included high-resolution 3D sagittal magnetization-prepared rapid acquisition gradient echo (MPRAGE) T1-weighted sequence, fast spin-echo (FSE) T2-weighted sequence, fluid-attenuated inversion recovery (FLAIR) sequence, susceptibility-weighted (SWI) sequence, and diffusion-tensor (DTI) sequence. All the MR scans were conducted in parallel with the anterior–posterior commissural plane using an echo planar imaging sequence.

### Imaging sequences

The scanning parameters were set as follows: MPRAGE: repetition time (TR) = 5000 ms, echo time (TE) = 2960 ms, flip angle = 12°, filed of view (FOV) = 256 × 256 mm^2^, matrix size = 256 × 256, slice thickness = 1 mm, voxel size = 1.0 × 1.0 × 1.0 mm. T2WI: TR = 3800 ms, TE = 99 ms, FOV = 220 × 220 mm^2^, slice thickness = 5 mm; FLAIR: TR = 8000 ms, TE = 81 ms, FOV = 220 × 220 mm^2^, slice thickness = 5 mm, matrix = 217 × 312; SWI: TR = 27 ms, TE = 20 ms, slices number = 64, slice thickness = 2.0 mm, voxel size = 0.9 × 0.9 × 2.0 mm; DTI: TR = 10,300 ms, TE = 95 ms, FOV = 256 × 256 mm^2^, matrix = 128 × 128, slice thickness = 2 mm, voxel size = 2.0 × 2.0 × 2.0 mm, number of direction = 64, b value = 1000 s/mm^2^, b0 value = 0 s/mm^2^. All scans were conducted at a minimum of 12 h since the last dose of anti-Parkinson’s drugs.

### Processing steps for EPVS volume fraction

Enlarged perivascular spaces are defined as spaces that surround and follow blood vessels that display signals resembling those of cerebrospinal fluid (CSF), exhibiting low signals on T1WI, high signals on T2 WI, and low signals on FLAIR images without diffusion restrictions. PVS volumes were evaluated in three brain regions on axial T2WI, including the slice with the largest number of PVS at the level of basal ganglia (BG), centrum semiovale (CSO), and lateral ventricle body (LVB) by two experienced neuroradiologists (with more than 5 years of experience) blinded to clinical information independently. The EPVS volume fraction was calculated as the ratio of PVS volume to the sum of gray matter volume and white matter volume, which were segmented using Computational Anatomy Toolbox in Statistical Parametric Mapping (SPM12).

### Processing steps for DTI-ALPS

**Fig. 2:**
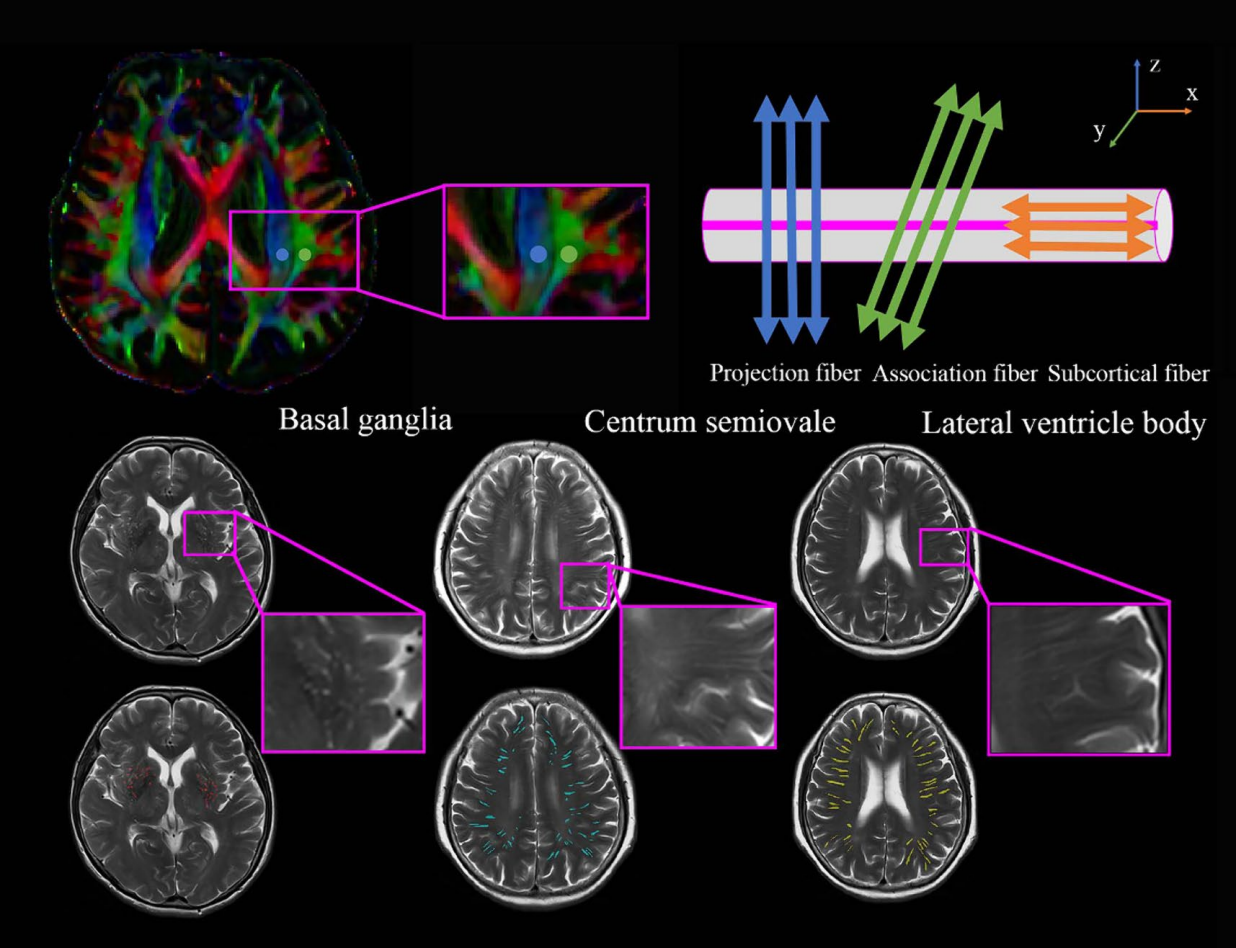
Schematic diagram of the DTI-ALPS index and PVS volume evaluation. (Upper) (left) ROIs on the projection fibers (blue) and association fibers (green), drawn on the colored FA map. (Right) spatial correlation between the perivascular space and subcortical fibers (red; *x*-axis), association fibers (green; *y*-axis), and projection fibers (blue; *z*-axis). (Below) T2-weighted images and PVS mask in the area of basal ganglia (left; red), centrum semiovale (middle, blue), and the level of lateral ventricle body (right, yellow).

The process for DTI-ALPS calculation is outlined in Fig. 2. The DTI-ALPS method allows for quantifying glymphatic activity along the perivascular space using multidirectional diffusivity maps obtained from DTI data. DTI images were processed and fitted using the FMRIB Software Library (FSL, http://www.fmrib.ox.ac.uk/fsl/), which includes the following steps: (1) denoising using MarchenkoPastur principal component analysis; (2) removal of Gibbs ringing due to partial *k*-space acquisition; (3) correction of motion and distortion artifacts from B0 inhomogeneities; (4) Eddy-currents correction; (5) correction of bias field. The output contains color-coded fractional anisotropy (colFA) and diffusivity maps in the *x*- and *y*- and *z*-axis (*D*_*xx*_, *D*_*yy*_, *D*_*zz*_). Spherical ROIs with a diameter of 5 mm were placed independently on colFA maps in the areas of bilateral projection and association fibers at the level of the lateral ventricle body. The placement was confirmed by SWI, which showed that the parenchymal vessels run laterally according to the previous study [24].

### Statistical methods

The clinical data of HC, PDD converters, and PDD nonconverters were compared using one-way analysis of variance (ANOVA), two sample *t* test, and the *χ*^2^ test, as appropriate. The inter-observer agreement on the diffusivity coefficient of DTI-ALPS and PVS measurements between the two readers was assessed using the inter-observer correlation coefficient (ICC). Differences in the diffusivity coefficient of the DTI-ALPS index and PVS among the three groups were compared using the Kruskal–Wallis *H* test followed by a post hoc Dunnett T3 test, under Bonferroni correction. Partial correlation analysis was performed to examine the relationship between DTI-ALPS, PVS volume fraction, and cognitive performance after adjustments for age, sex, and years of education. The mediation effects of PVS fraction on the association between DTI-ALPS and cognitive performance were further assessed. Furthermore, the significant diffusion coefficient of the DTI-ALPS index and the EPVS alterations between PDD converters and PDD nonconverters were further measured by receiver operating curve (ROC) analyses. The optimal cut-off value was determined by maximizing Youden’s index, and specificity, sensitivity, and area under the ROC (AUC) were presented.

A two-tailed *P* < 0.05 was considered statistically different. Statistical analyses were performed using SPSS software (version 26; IBM-SPSS) and R software (version 3.5.1; R Foundation for Statistical Computing, Vienna, Austria).

## Results

### Demographics and clinical assessment

The demographic characteristics of PDD converters, PDD nonconverters, and HC are summarized in Table 1. No significant differences in age, sex, or years of education are noticed among the three groups. PDD converters showed higher UPDRSIII score compared to PDD nonconverters (31.55 ± 8.96 vs 26.03 ± 7.61, *P* = 0.02). However, there were no significant differences in the disease duration, H–Y stage, and LEDD dosage between PDD converters and PDD nonconverters.

**Table 1:** Demographic and clinical characteristics of participants

	PDD converter (*n* = 29)	PDD nonconverters (*n* = 35)	HC (*n* = 35)	*P*
Age at onset	63.87 ± 5.32	65.77 ± 3.86	62.94 ± 5.22	0.05
Sex (male/female)	16/13	18/17	16/19	0.67
Education (years)	9.52 ± 1.84	8.86 ± 2.07	9.03 ± 1.89	0.39
Duration	4.83 ± 1.71	4.11 ± 1.60	–	0.11
H–Y stage	2.48 ± 1.02	2.34 ± 1.00	–	0.61
UPDRSIII	31.55 ± 8.96	26.03 ± 7.61	–	0.02*
LEDD (mg)	350.66 ± 123.11	370.83 ± 122.96	–	0.57
Cognitive performance at baseline				
MMSE	23.45 ± 1.64	23.00 ± 1.50	29.00 ± 0.77	< 0.001***^,b,c^
MoCA	21.17 ± 1.63	21.54 ± 1.40	27.60 ± 1.09	< 0.001***^,b,c^
BNT	21.98 ± 1.86	21.71 ± 1.69	25.14 ± 1.70	< 0.001***^,b,c^
DST	9.97 ± 1.68	11.37 ± 2.07	13.86 ± 1.96	< 0.001***^,a,b,c^
RAVLT IR	28.34 ± 3.35	29.94 ± 3.76	45.43 ± 4.17	< 0.001***^,b,c^
RAVLT PF	29.07 ± 4.64	28.00 ± 3.65	21.51 ± 2.25	< 0.001***^,b,c^
TMT	99.38 ± 12.97	91.51 ± 9.68	29.97 ± 4.78	< 0.001***^,a,b,c^
CDT	4.76 ± 1.24	5.34 ± 1.43	9.23 ± 0.74	< 0.001***^,b,c^
CFT	26.69 ± 2.44	27.63 ± 2.20	33.29 ± 2.57	< 0.001***^,b,c^
CCT	10.45 ± 1.70	10.46 ± 1.40	12.80 ± 1.51	< 0.001***^,b,c^
Cognitive performance at follow-up				
MMSE	12.00 ± 3.15	22.51 ± 1.62	–	< 0.001***
MoCA	11.03 ± 1.97	21.20 ± 1.69	–	< 0.001***
BNT	22.90 ± 2.34	22.49 ± 2.28	–	0.48
DST	9.10 ± 1.70	10.74 ± 1.80	–	< 0.001***
RAVLT IR	26.83 ± 2.71	28.51 ± 3.18	–	0.03*
RAVLT PF	31.03 ± 4.55	28.86 ± 3.45	–	0.05
TMT	113.55 ± 21.96	95.94 ± 11.85	–	< 0.001***
CDT	3.55 ± 1.30	5.20 ± 1.32	–	< 0.001***
CFT	26.14 ± 2.61	27.11 ± 2.01	–	0.10
CCT	10.14 ± 1.62	10.49 ± 1.50	–	0.38

Results are expressed as mean ± standard deviation

*HC* healthy control, *PD* Parkinson’s disease, *H–Y stage* Hoeh and Yahr stage, *UPDRSIII* Unified Parkinson’s Disease Rating Scale Part III, *MMSE* Mini-mental state examination, *MoCA* Montreal Cognitive Assessment, *LEDD* levodopa equivalent daily dose, *BNT* Boston Naming Test, *DST* Digital symbol test, *RAVLT IR* Rey Auditory Verbal Learning Test RAVLT PF, *TMT* Trail Making Test, *CDT* Clock Drawing Test, *CFT* Rey Complex Figure Test, *CCT* Clock Copying Test

*P < 0.05, **P < 0.01, ***P < 0.001

^a^PDD converters vs PDD nonconverters

^b^PDD converters vs HC

^c^PDD nonconverters vs HC in post hoc analysis

### DTI-ALPS index in PD-MCI patients and control subjects

**Fig. 3:**
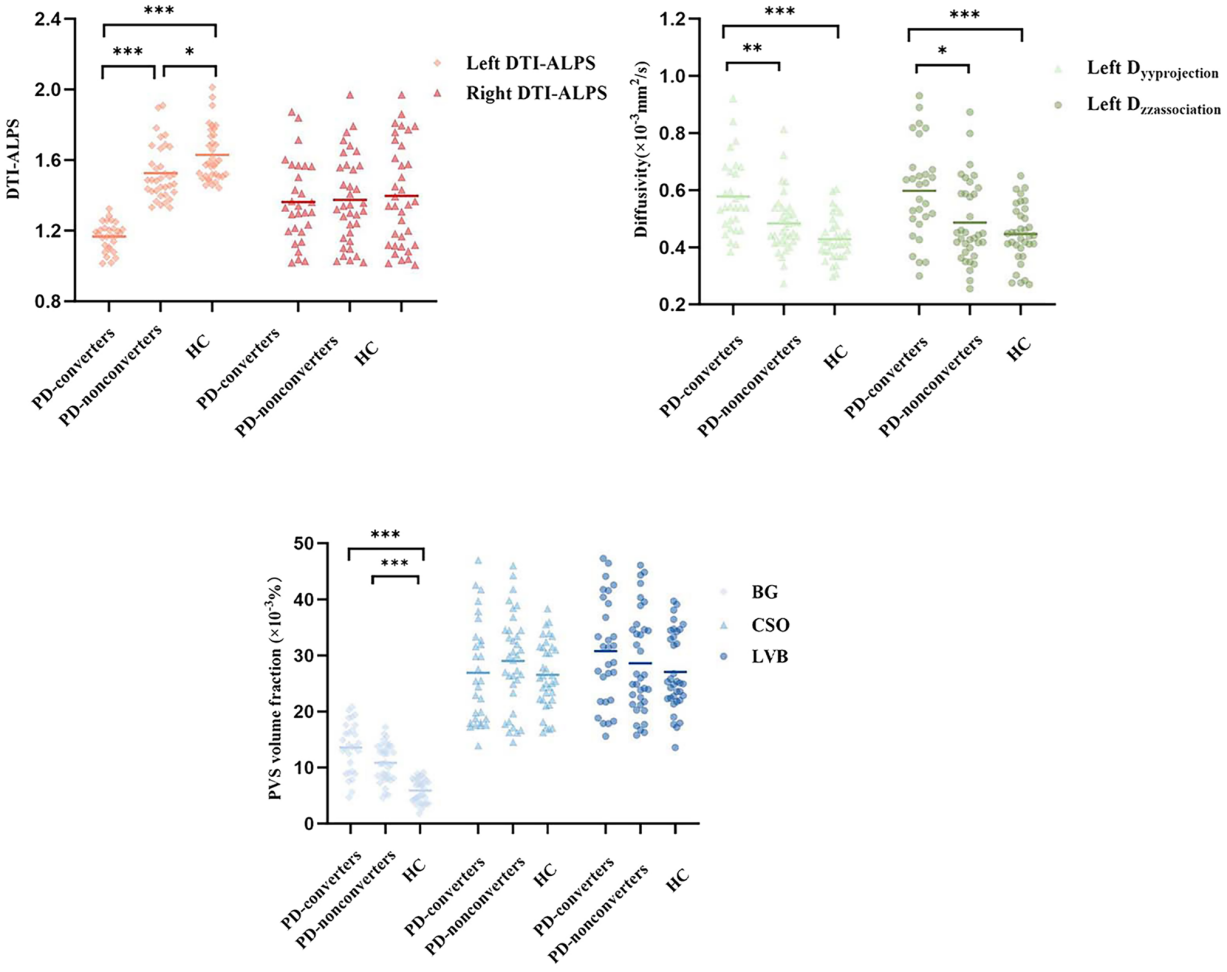
Differences in left DTI-ALPS, diffusivities, and PVS volume fraction among HC, PDD converters, and PDD nonconverters using the Kruskal–Wallis *H* test followed by a post hoc Dunnett T3 test. *P < 0.05, **P < 0.01, ***P < 0.001, statistically significant under false discovery rate adjustment

The measure of DTI-ALPS index showed excellent inter-observer agreement (ICC for right ALPS index, 0.94 [95% CI 0.92, 0.96]; ICC for left ALPS index, 0.92 [95% CI 0.88, 0.94]). The median left ALPS index was lower in both PDD converters (1.18) and PDD nonconverters (1.49) compared with HCs (1.58) (*P* < 0.001), as well as between PDD converters and PDD nonconverters (*P* = 0.02). Meanwhile, diffusivity along the *y*-axis in the left projection neural fibers, as well as diffusivity along the *z*-axis in the left association neural fibers was higher in PDD converters (0.54, 0.62, respectively) compared to HCs (0.42, 0.44, respectively) and PDD nonconverters (0.45, 0.44, respectively) (*P* < 0.001, *P* < 0.05, respectively). (Table 2, Fig. 3).

**Table 2:** Diffusion coefficient of the DTI-ALPS index in each direction among the PDD converters, PDD nonconverters, and HC

Diffusion coefficient	PDD converters (*n* = 29)	PDD nonconverters (*n* = 35)	HC (*n* = 35)	*P*	Post hoc tests *P* value		
					PDD converters vs HC	PDD nonconverters vs HC	PDD converters vs PDD nonconverters
Left *D*_*xx*assoc_ (× 10^−3^mm^2^/s)	0.67 (0.55–0.83)	0.76 (0.65–0.76)	0.79 (0.70–0.87)	0.02*	0.02*	0.52	0.25
Left *D*_*xx*proj_ (× 10^−3^mm^2^/s)	0.61 (0.54–0.72)	0.66 (0.58–0.73)	0.61 (0.56–0.72)	0.24	–	–	–
Left *D*_*yy*assoc_ (× 10^−3^mm^2^/s)	0.92 (0.78–1.02)	0.83 (0.74–0.95)	0.83 (0.73–0.95)	0.63	–	–	–
Left *D*_*yy*proj_ (× 10^−3^mm^2^/s)	0.54 (0.47–0.67)	0.45 (0.42–0.54)	0.42 (0.37–0.47)	< 0.001***	< 0.001***	0.06	0.002**
Left *D*_*zz*assoc_ (× 10^−3^mm^2^/s)	0.62 (0.49–0.67)	0.44 (0.38–0.59)	0.44 (0.40–0.53)	0.001**	< 0.001***	0.45	0.03*
Left *D*_*zz*proj_ (× 10^−3^mm^2^/s)	0.89 (0.78–1.02)	0.90 (0.81–0.96)	0.95 (0.87–1.04)	0.08	–	–	–
Right *D*_*xx*assoc_ (× 10^−3^mm^2^/s)	0.75 (0.67–0.90)	0.77 (0.67–0.87)	0.78 (0.70–0.91)	0.97	–	–	–
Right *D*_*xx*proj_ (× 10^−3^mm^2^/s)	0.23 (0.20–0.25)	0.20 (0.18–0.23)	0.21 (0.18–0.22)	0.08	–	–	–
Right *D*_*yy*assoc_ (× 10^−3^mm^2^/s)	0.92 (0.81–1.05)	0.92 (0.77–1.01)	0.88 (0.78–0.98)	0.46	–	–	–
Right *D*_*yy*proj_ (× 10^−3^mm^2^/s)	0.23 (0.21–0.26)	0.20 (0.18–0.23)	0.21 (0.18–0.23)	0.05	–	–	–
Right *D*_*zz*assoc_ (× 10^−3^mm^2^/s)	0.51 (0.38–0.62)	0.51 (0.41–0.66)	0.51 (0.41–0.59)	0.96	–	–	–
Right *D*_*zz*proj_ (× 10^−3^mm^2^/s)	0.22 (0.20–0.25)	0.20 (0.18–0.23)	0.20 (0.18–0.22)	0.08	–	–	–
Left ALPS index	1.18 (1.10–1.21)	1.49 (1.42–1.67)	1.58 (1.51–1.74)	< 0.001***	< 0.001***	0.02*	< 0.001***
Right ALPS index	1.33 (1.20–1.57)	1.34 (1.16–1.57)	1.35 (1.12–1.68)	0.92	–	–	–

Results are expressed as median [interquartile range (IQR)]

*HC* healthy control, *PD* Parkinson’s disease, *D*_*xxassoc*_ diffusivity along the *x*-axis in association fiber area, *D*_*yyassoc*_ diffusivity along the *y*-axis in association fiber area, *D*_*zzassoc*_ diffusivity along the *z*-axis in association fiber area, *D*_*xxproj*_ diffusivity along the *x*-axis in projection fiber area, *D*_*yyproj*_ diffusivity along the *y*-axis in projection area, *D*_*zzproj*_ diffusivity long the *z*-axis in projection area, *ALPS* analysis along the perivascular space

*P < 0.05; **P < 0.01; ***P < 0.001 under false discovery rate adjustment

### PVS volume fraction in PD-MCI patients and control subjects

The measure of PVS volume fraction showed excellent inter-observer agreement (ICC for BG-PVS, 0.85 [95% CI 0.78, 0.90]; ICC for CSO-PVS, 0.89 [95% CI 0.84, 0.93]; ICC for LVB-PVS, 0.87 [95% CI 0.80, 0.93]). Significant differences were observed in BG-PVS volume fraction among the three groups (*P* < 0.001). Compared with HCs (6.2), PDD converters (13.7) and PDD nonconverters (10.7) showed a significantly larger BG-PVS volume fraction (*P* < 0.001). However, there were no significant differences in the CSO-PVS (*P* = 0.38) or LVB-PVS (*P* = 0.22) volume fraction among the three groups (Table 3, Fig. 3).

**Table 3:** Vascular burden on neuroimaging of PDD converters, PDD nonconverters, and HC

PVS volume fraction (× 10^–3^%)	PDD converters (*n* = 29)	PDD nonconverters (*n* = 35)	HC (*n* = 35)	*P*	Post hoc tests *P* value		
					PDD converters vs HC	PDD nonconverters vs HC	PDD converters vs PDD nonconverters
BG-PVS	13.7 (9.2–17.1)	10.7 (8.1–13.7)	6.2 (4.1–7.7)	< 0.001***	< 0.001***	< 0.001***	0.03*
CSO-PVS	25.5 (18.4–33.0)	29.9 (23.4–34.5)	26.0 (22.1–31.5)	0.38	–	–	–
LVB-PVS	31.4 (21.9–39.8)	26.0 (21.3–34.6)	25.1 (22.3–34.3)	0.22	–	–	–

Results are expressed as median [interquartile range (IQR)]

*HC* healthy control, *PD* Parkinson’s disease, *BG-PVS* perivascular space in the basal ganglia, *CSO-PVS* perivascular space in the centrum semiovale, *LVB-PVS* perivascular space in the lateral ventricle body

*P < 0.05; **P < 0.01; ***P < 0.001 under false discovery rate adjustment

PVS volume fraction = PVS volume/(gray matter volume + white matter volume) × 100%

### Relationship between PVS volume fraction and DTI-ALPS index and cognitive impairment

**Fig. 4:**
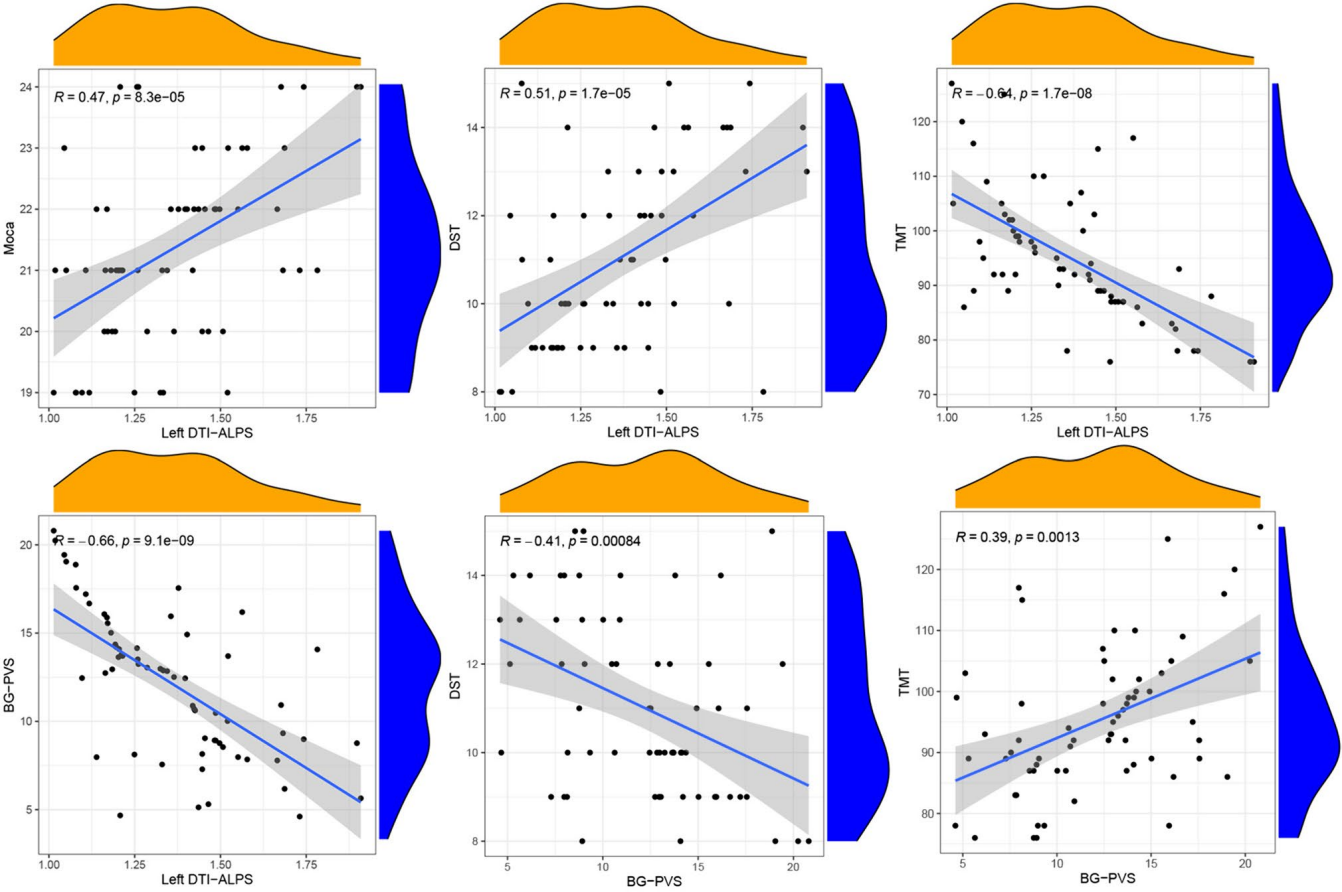
Partial correlations among the left DTI-ALPS index, BG-PVS volume fraction, and cognitive performance after adjustments for age, sex, and years of education

The DTI-ALPS index in the left hemisphere showed significant correlation with global cognitive performance (*r* = 0.47, *P* < 0.001), Digit Span Test (*r* = 0.51, *P* < 0.001), and Trail Making Test (*r* = − 0.64, *P* < 0.001) in PD-MCI group. Furthermore, BG-PVS volume fraction was significantly correlated with Digit Span Test (*r* = 0.39, *P* < 0.001), as well as Trail Making Test (*r* = − 0.41, *P* < 0.01) in PD-MCI group (Fig. 4). There was no statistically significant correlation between DTI-ALPS index, as well as BG-PVS, and cognitive performance in the HC group.

Partial correlation analyses showed a significant negative correlation between the DTI-ALPS index and BG-PVS volume fraction in PD-MCI patients (*r* = − 0.66, *P* < 0.001), after controlling for age, sex, and years of education (Fig. 4). However, no significant correlation was found between the DTI-ALPS index and COS-PVS or LVB-PVS volume fraction. Mediation analysis indicated that BG-PVS did not have a significant indirect effect on the relationship between DTI-ALPS and global cognitive performance (*β* = − 0.01, *P* = 0.93), Digit Span Testing (*β* = 0.80, *P* = 0.52), as well as Trail Making Testing (*β* = − 2.48, *P* = 0.59).

### Predictive value of glymphatic function for dementia conversion

**Fig. 5:**
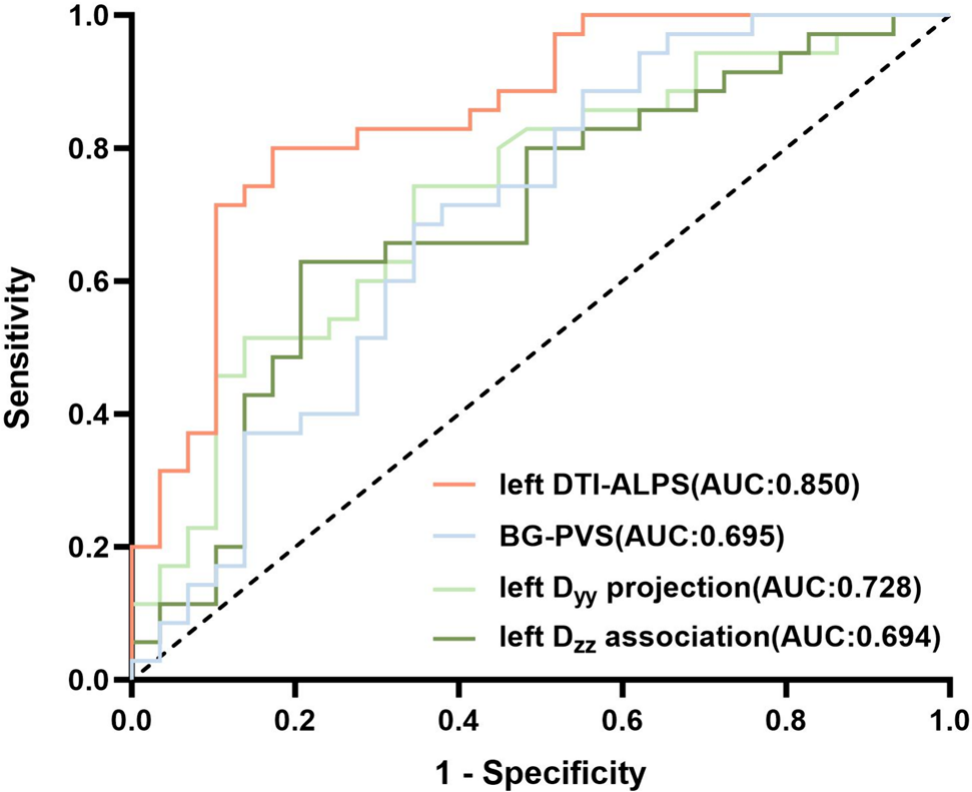
ROC analyses of the left DTI-ALPS index, diffusivities, and BG-PVS

The DTI-ALPS in the left hemisphere exhibited the optimal performance in distinguishing between PDD converters and PDD nonconverters (AUC 0.850; 95% confidence interval [CI] 0.754–0.946, *P* < 0.001), followed by the left *D*_*yy*_ projection index (AUC 0.728; 95% CI 0.603–0.853, *P* = 0.002). The left *D*_*zz*_ association index and the PVS of basal ganglia showed moderate predictive value in differentiating PDD converters and PDD nonconverters (AUC 0.694, 95% CI 0.561–0.826; AUC 0.695, 95% CI 0.561–0.828, respectively) (Table 4, Fig. 5).

**Table 4:** ROC analyses of glymphatic function index in differentiating PDD converters and PDD nonconverters

Glymphatic function index	AUC	*P* value	95% CI	Sensitivity	Specificity	Cut-off point
Left *D*_*yy*proj_	0.728	0.002**	0.603–0.853	0.655	0.743	0.398
Left *D*_*zz*assoc_	0.694	0.008**	0.561–0.826	0.793	0.629	0.422
Left ALPS	0.850	< 0.001***	0.754–0.946	0.800	0.828	0.628
BG-PVS	0.695	0.008**	0.561–0.828	0.655	0.686	0.341

*AUC* area under the curve, *CI* confidence interval, *D*_*yyproj*_ diffusivity along the *y*-axis in projection area, *D*_*zzassoc*_ diffusivity along the *z*-axis in association fiber area, *Left ALPS* analysis along the perivascular space in the left hemisphere, *BG-PVS* perivascular space in the basal ganglia

## Discussion

In the present study, we explored in vivo glymphatic dysfunction in a cohort of PD-MCI patients to predict the dementia conversion during the follow-up period. We demonstrated that PD patients with MCI who progressed to dementia exhibited reduced glymphatic activity and enlarged PVS in the basal ganglia in comparison to those nonconverters, and the glymphatic dysfunction was associated with enlarged PVS volume fraction, global cognitive performance, and executive function. Furthermore, the DTI-ALPS index can well identify PDD converters from nonconverters in patients with PD and MCI. Therefore, glymphatic impairment may contribute to the conversion from MCI to dementia in PD, and is likely to be a promising indicator to assist in the dementia prediction and potential neuroprotective strategy for slowing the cognitive deteriorative process in PD.

The assessment of glymphatic function in vivo has been hampered by the lack of direct imaging measures not requiring the injection of contrast agent. To overcome this obstacle, DTI-ALPS index was recently introduced to assess diffusivity along the perivascular space in vivo and hence, as measure of brain glymphatic function. Researchers reported that there is a negative correlation between α-synuclein deposition and AQP4 expression in the brain of PD patients, indicating a link between glymphatic dysfunction and pathologic protein accumulation [25]. This also supports the hypothesis of “prion-like propagation”, where a drainage system like the glymphatic system helps clear the accumulation of toxic proteins or, in the case of glymphatic system failure could contribute to the neurodegeneration and brain pathology [26]. The development of dementia in PD is thought to be a culminating result of the a heavy burden of α-synuclein pathology in limbic and neocortical structures [27], indicating that dementia is related to a more severe glymphatic impairment. Previous studies have also identified the relationship between cognitive impairment and glymphatic dysfunction in schizophrenia [28], AD [29], frontotemporal dementia [30], and PD [14]. Therefore, in accordance with prior studies, our findings also found the reduced glymphatic activity in the patients with MCI who develop dementia and cognitive deterioration. Considering the elevated incidence of dementia in PD patients, a multitude of researchers have been dedicated to identify MRI biomarkers to be indicative of cognitive deterioration in PD in order to facilitate an early identification and prevention. For example, researchers have proposed using cortical thickness on T1WI [31], and white matter structural connectivity on DTI [32] as predictors for PD cognitive progression. Hence, glymphatic dysfunction, assessed by DTI-ALPS, could be used as an additional MRI indicator for predicting cognitive deterioration, and also as a potential supplement for the intervention of cognitive function in patients with PD.

In our study, PDD converters were associated with glymphatic dysfunction in the left hemisphere, but not in the right hemisphere. One possible explanation could be that glymphatic impairments begins in the left hemisphere for right-handed patients as evidenced by motor asymmetry in PD [33, 34]. In line with our study, previous research identified abnormalities in the left-hemispheric DTI-ALPS index in PD [19]. However, Qin et al. investigated the correlation between glymphatic function and motor symptoms, suggesting the right hemispheric DTI-ALPS index was lower than the left side [21]. Therefore, the debate regarding whether PD patients exhibited more pronounced glymphatic impairment in the left hemisphere persists, necessitating further investigation with subgroup analysis. Apart from the DTI-ALPS index in the left hemisphere, PDD converters also exhibited higher diffusivity along the *y*-axis in the projection neural fibers and diffusivity along the *z*-axis in the association neural fibers in the left hemisphere. Since diffusivity in the *y*-axis and *z*-axis does not align with the perivascular water flow, therefore, the different diffusivity in the projection and association fibers could be attributed to white matter degeneration due to cognitive deterioration in the projection or association fibers as indicated in previous studies [35, 36].

The perivascular space is a region that surrounds arterioles, capillaries, and venules in the brain, and is considered as a part of glymphatic system both structurally and functionally [37]. Animal studies reported diminished glymphatic influx and heightened perivascular α-syn aggregation, as well as blocked glymphatic efflux and increased deposition of α-syn and worsened PD pathology [6].Therefore, glymphatic dysfunction may contribute to the dilation of PVS, which in turn lead to the aggregation and accumulation of misfolded proteins.

Conversely, an elevated PVS burden could further exacerbate the glymphatic dysfunction. Among the three classic locations of PVS burden according to previous reports, only PVS burden in the basal ganglion is significantly enlarged, representing a heavy neurovascular burden in the BG region linked to cognitive progression. Our findings align with recent research showing an elevated presence of PVS in PD patients, with EPVS in the BG region likely more representative [12, 17, 38]. Researchers have proposed that dementia is more strongly associated with visible PVS in the BG than with PVS at other sites [37]. Furthermore, functional and structural alterations in the BG region have been related to the progression of PD pathology and its clinical manifestations [39, 40]. As a result, the heavy vascular burden in BG is linked to cognitive deterioration in patients with PD and MCI.

Besides, both DTI-ALPS index and BG-PVS showed significant correlation with global cognitive performance and the executive function. In line with our findings, DTI-ALPS index and EPVS in BG were reported to be closely related to cognitive function, information processing, as well as executive function [21, 41, 42].Nevertheless, several reports are in contrast to our findings, reporting no statistical correlation between BG-EPVS and cognition [10]. The difference in the definition of the PVS score may be one of the contributing factors. PVS scoring based on the number of EPVS may result in a higher rate of severe vascular burden. Despite the significant correlations between DTI-ALPS, BG-EPVS, and cognitive function, our study did not find statistical mediation effect of BG-EPVS on the relationship between DTI-ALPS and cognitive function. One possible explanation could be that the EPVS in the BG serves as an independent factor influencing cognition [41]. It is closely associated with lacunar infarcts and periventricular white matter hyperintensities, which can directly impact cognitive and executive function by interrupting the prefrontal subcortical circuit [43]. In contrast to our findings, a recent study found that DTI-ALPS acted as a mediator in the relationship between white matter hyperintensities and cognitive performance [44]. Therefore, the potential causal relationship between EPVS, glymphatic dysfunction, and cognition needs further investigation.

This study had several limitations. First, the current study had a small sample size in terms of longitudinal follow-up time. Future studies with a larger sample size and a longer follow-up period are needed to confirm our results. Second, although correlations between glymphatic dysfunction and cognitive deterioration over time were initially established, the causal effect between glymphatic impairment and cognitive conversion during period time was not determined in the current study. Third, the ROIs for the DTI-ALPS calculations were manually delineated, and the PVS was defined by fractional volume in the three specified regions. Despite having perfect inter-observer and intra-observer reliability, utilizing automated or semi-automated delineation methods could reduce potential bias and improve the clinical utility. In addition, future studies are needed to explore the influence of additional factors, such as white matter hyperintensities, on the DTI-ALPS index and cognitive decline in PD patients. Despite the limitations mentioned above, the DTI-ALPS index, as a non-invasive imaging method, still holds potential for investigating glymphatic system function in vivo, and therefore, serves as imaging indicator in forecasting progress to dementia in PD-MCI stage.

Our study found a lower DTI-ALPS in patients with PD and MCI who develop dementia, indicating potential dysfunction in the glymphatic system. Besides, DTI-ALPS index was correlated with enlarged BG-PVS and global cognitive performance, as well as executive function. Therefore, the DTI-ALPS may assist in identifying PD with MCI at a high risk of cognitive deterioration before the onset of dementia, thereby providing potential therapeutic strategies.

## Data Availability

The datasets used and analyzed during this study are available from the corresponding author on reasonable request.

## Declarations

### Guarantor

The scientific guarantor of this publication is Dr. Guoguang Fan. The authors confirm that all methods were carried out in accordance with relevant guidelines and regulations.
